# Anti-N-methyl-D-Aspartate Receptor Encephalitis Mimicking Sporadic Creutzfeldt–Jakob Disease

**DOI:** 10.3389/fneur.2020.593680

**Published:** 2020-11-26

**Authors:** Jiao Liu, Liyan Chen, Jing Yang, Lan Wang, Huifang Shang, Xueping Chen

**Affiliations:** ^1^Department of Neurology, West China Hospital, Sichuan University, Chengdu, China; ^2^Department of Neurology, Chongqing People's Hospital, Chongqing, China

**Keywords:** anti-NMDAR encephalitis, sporadic Creutzfeldt-Jakob disease, 14-3-3 protein, differential diagnosis, immunomodulatory therapy

## Abstract

**Objectives:** Anti-N-methyl-D-aspartate receptor (NMDAR) encephalitis and sporadic Creutzfeldt–Jakob disease (sCJD) share similar clinical features. Here, we present two unusual cases of anti-NMDAR encephalitis who were misdiagnosed as sCJD at first.

**Methods:** We described two patients' clinical manifestations, as well as the string of symptomatological evolution, treatments, and follow-up results.

**Results:** Our patients presented with rapidly progressive dementia, memory problems, psychiatric symptoms, and movement disorders, and we considered all these symptoms as a presenting feature of sCJD at first, but the cerebrospinal fluid examination showed positive results for both the 14-3-3 protein and antibodies against NMDAR. Immunomodulatory treatment led to a resolution of these deficits, and both of them remained in remission after treatment.

**Conclusion:** Anti-NMDAR encephalitis can present with rapidly progressive cognitive decline, and sometimes laboratory investigations can be misleading. The examination for the presence of NMDAR antibodies is necessary, even with the presence of 14-3-3 protein. Early immunomodulatory therapy should be considered, especially for patients with high titers of NMDAR antibodies.

## Introduction

Anti-N-methyl-D-aspartate receptor (NMDAR) encephalitis and sporadic Creutzfeldt–Jakob disease (sCJD) share a lot of similar clinical features, such as prominent cognitive decline, memory problems, psychiatric symptoms, and movement disorders (1). Recent studies reported that some patients confirmed to have sCJD had low levels of serum antibodies to NMDAR, glycine receptor, and voltage-gated potassium channel (VGKC) complex (2–4). Conversely, some researchers have found that some forms of autoimmune encephalitis could be misdiagnosed as CJD, including Hashimoto's and VGKC complex antibody-associated encephalopathies (5, 6). In addition, Grau-Rivera et al. also reported that some patients with a diagnosis of immunotherapy-responsive limbic encephalitis had their treatment delayed because of a suspected diagnosis of sCJD (7). Therefore, the shared clinical features complicate the differential diagnosis of these disorders.

Here, we described two patients first diagnosed with suspected sCJD presenting with rapidly progressive dementia, but the cerebrospinal fluid (CSF) examination showed positive results for both the 14-3-3 protein and antibodies against NMDAR. Furthermore, the follow-up findings confirmed the diagnosis of anti-NMDAR encephalitis.

## Report of Two Cases

### Case 1

A 46-year-old male deliveryman was admitted to the hospital in March 2018 with a 4-month history of progressive memory impairment. Four months before admission, he developed changes characterized by memory impairment; he could not remember where to deliver. He lost his quick intellect and responded slowly to certain situations. He did not talk much and was unable to say the right words at the right time. One month before admission, his symptoms became worse. He could not calculate correctly and complained of muscle trembling in the limbs. Then, a feeling of heaviness in his legs and double vision precipitated his admission. He had a history of type 2 diabetes mellitus. On admission, the patient was oriented, but his speech was slurred, with a Mini-Mental State Examination (MMSE) score of 14 of 30. He performed poorly on tests of cognitive function. Impairment was greatest for delayed and verbal recognition memory, and executive functions, language, and visuospatial functions were also impaired. Myoclonic jerks of the upper limbs were observed, with hypomimia and parkinsonian gait. During the next 1 month, he became increasingly confused, with difficulty to recognize family members. He acted like a child and needed to be taken care of. He could not wear socks independently and had difficulties putting on different pieces of clothing, but his motor function was normal. While hospitalized, routine hematological and biochemical examinations were normal. A vasculitis screen based on serological markers was negative. Diffusion-weighted imaging performed 5 months after onset did not show specific abnormality. An electroencephalogram (EEG) showed a mild slowing of the background activity, without periodic sharp wave complexes. CSF analysis demonstrated an elevated protein level (550 mg/dl) and increased cell count (17 × 10^6^/L) with a lymphocytic predominance. The results of a thorough infectious workup conducted in CSF and serum were negative. The 14-3-3 protein was strongly positive in CSF. He was homozygous for methionine at prion protein gene codon 129, and prion protein gene sequencing revealed no mutations. A neurological autoantibody panel for serum and CSF revealed no evidence of antibodies to VGKC (LGI1/ Caspr2), GABAbR, GAD65, AMPA1R, AMPA2R, Hu, Ri, Yo, Ma2, CV2/CRMP5, and amphiphysin. However, CSF antibodies to the NMDAR were positive (1:100). In the presence of positive NMDAR antibodies, immunosuppressant treatment with intravenous immunoglobulin (IVIG) was commenced 18 days after admission. After 5 days of intravenous IVIG therapy, his cognitive performance improved, with an MMSE score of 19/30. Unfortunately, his family refused further treatment, and immunosuppressive treatment due to the economic problems, including high-dose intravenous corticosteroids, plasmapheresis, and cyclophosphamide, was discontinued. By 12 months from onset, there was a remarkable clinical improvement, and he only had slight acalculia. Eighteen months after the first neurological examination, he was alert, and the MMSE score was 24/30, during which he was unable to perform the serial-7 calculations. He was able to help his son to do the delivery work. During the follow-up (we followed him every 3 months, and the most recent follow-up was performed in April 2020), he remained in remission without any further treatment.

### Case 2

**Figure 1:**
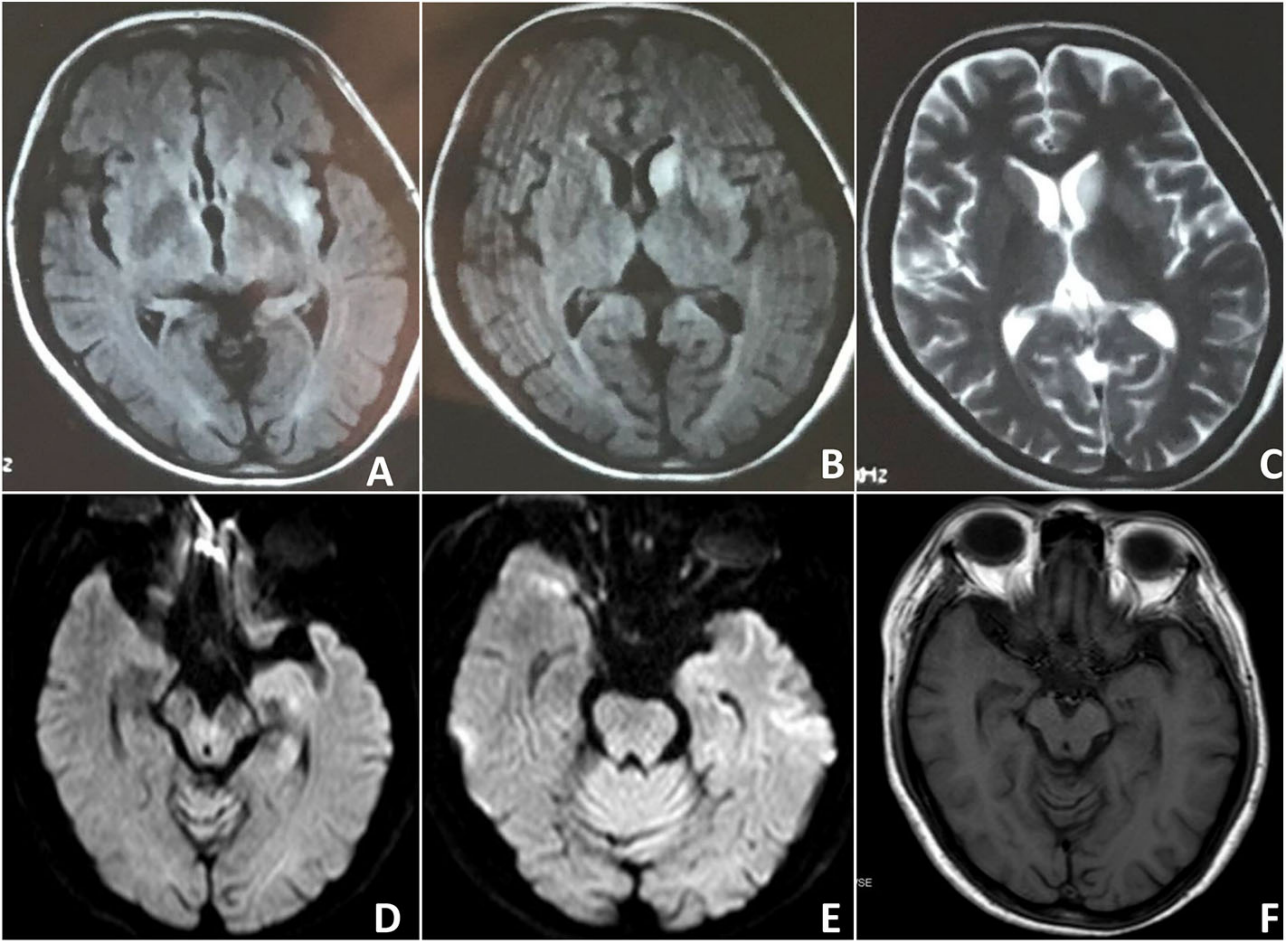
Case 2: Findings on the brain MRI. Initial brain MRI revealed asymmetric FLAIR and T2 hyperintensities in the putamen and caudate. **(A–C)** Subsequent brain MRI showed bilateral hippocampal volume reduction **(D–F)**.

A 46-year-old woman was admitted to the hospital in June 2018. Three months before admission, she complained about headache and dizziness, with mild memory impairment. One month before admission, her symptoms became noticeably worse, and her family noted her behavioral disturbances and rapidly progressive cognitive impairment. She was unable to complete her sentences and recognize people she used to be familiar with. Incoherent thoughts, disorganized speech, and catatonic behavior precipitated her admission to the local hospital. Initial brain MRI in the local hospital revealed asymmetric FLAIR and T2 hyperintensities in the putamen and caudate (Figure 1). However, her symptoms were not relieved after admission, and the symptoms were followed by sleep rhythm changes, characterized by irritability at night and apathy during daytime. Her daughter noticed that she was out of breath for no reason occasionally. Motor tasks became difficult, and she could no longer perform agricultural activity. Then, she was transferred to our hospital, and she had signs of bradyphrenia, word-finding difficulties, and shuffling gait on admission. Her memory, insight, and judgment were abnormal, accompanied with apraxia and acalculia, with an MMSE score of 12 of 30. The muscle tone and strength were normal, but extensor plantar responses were bilaterally present. After admission, routine blood analysis and EEG were given, and the results were normal. Brain MRI performed in our hospital 5 months after onset showed bilateral hippocampal volume reduction and hyperintensities within the bilateral hippocampus on T2 FLAIR images. The routine CSF analysis was normal, including cell count, protein level, and glucose level, but the 14-3-3 protein was positive in CSF. The autoimmune panel was also ordered in both serum and CSF, and only antibodies to the NMDAR were detected in CSF (1:100). Contrast CT scans of the chest/abdomen/pelvis were negative for malignancies. Prion protein gene sequencing revealed methionine homozygosity at codon 129. Twelve days after admission, we treated the patient with intravenous IVIG and followed by intravenous methylprednisolone; the patient's neurological condition was improved obviously. After discharge, she received pulsed corticosteroid treatment for 3 months, and she obtained symptomatic relief, with an MMSE score of 17/30. By 8 months from onset, the symptoms of the patient relieved significantly, including cognitive and motor performance. She could recognize her family members and perform some agricultural activity and basic activities of daily living. Eighteen months after the first neurological examination, there was no obvious fluctuation of her symptoms, and the MMSE score was 22/30. She remained in remission at the time of the last follow-up performed 2 years after disease onset.

## Discussion

This article illustrates clinical presentations of anti-NMDAR encephalitis that mimic sCJD and demonstrates the potential for misdiagnosis of a treatable condition for a terminal one. The clinical manifestations of these two cases are compatible with sCJD, and they both present symptoms of rapidly progressive dementia. Adding to the complexity of diagnosis is the concurrence of anti-NMDAR antibodies in sCJD. In serum, Fujita et al. reported antibodies against the GluN2B subunit of NMDAR in 13 sCJD cases. Among these cases, six were diagnosed with definite sCJD, six were diagnosed with probable sCJD, and one was diagnosed with possible sCJD, but the clinical features were not clearly outlined in these patients (2). Mackay et al. found that two definite sCJD cases had low levels of serum anti-NMDAR antibodies (3). In CSF, antibodies against NMDAR with low titers were detected in an sCJD patient, and the diagnosis was validated by positive real-time quaking-induced conversion (RT-QUIC) results (8). Grau-Rivera et al. found that anti-NMDAR antibodies were detected in CSF of 1/346 (0.29%) patients with rapid neurologic deterioration suggestive of CJD, but this patient did not fulfill the diagnostic criteria for probable or possible CJD, whereas none of the 49 patients with definite CJD had anti-NMDAR antibodies in CSF (7). Furthermore, Maat et al. identified the neuropathologist who diagnosed autoimmune encephalitis in 22 of 181 patients with suspected CJD, and only one patient with possible CJD had CSF autoantibodies directed against NMDAR (9). Therefore, only a low number of patients with suspected sCJD had detectable NMDAR antibodies. However, when the laboratory findings are unavailable and non-diagnostic, immunological therapies may be applied because of the potential benefit. Interestingly, a previous study also showed that a series of patients with suspected autoimmune encephalitis-mediated dementia had a good response to immunotherapy, but almost 9% of them had been initially diagnosed as sCJD (5). The results of our study raised some critical issues. First, our case highlights the heterogeneity of anti-NMDAR encephalitis and supports diagnostic awareness in cases of rapidly progressive dementia. Biochemical overlap and antibody testing may cause further diagnostic confusion, but therapeutic possibilities should be considered. Our cases received immunosuppressive treatment with diagnostic possibilities of anti-NMDAR encephalitis and suspected sCJD, and both of them have shown a good response to immunotherapy. Second, higher levels of anti-NMDAR antibodies are very likely to be associated with an autoimmune encephalitis diagnosis. By contrast, Rossi et al. reviewed 150 sCJD cases and found 2 (1.3%) patients who developed serum antibodies to NMDAR antigens but only at low titers (10). Our cases were identified with high anti-NMDAR antibody titers (1:100), and they were finally diagnosed with anti-NMDAR encephalitis, but it remains important to consider sCJD in the differential diagnosis.

The clinical manifestations of our two cases include progressive cognitive/psychic/motor symptoms. According to the World Health Organization diagnostic criteria for sporadic Creutzfeldt–Jakob disease, these two cases were diagnosed with probable sCJD at first. We considered all these symptoms as a recognized and presenting feature of sCJD, and the differential diagnosis was supported by the increased levels of 14-3-3 protein in the CSF. However, the absence of EEG findings of sCJD poses additional diagnostic challenges. For case 1, the diagnosis of probable anti-NMDAR encephalitis can be made according to Graus et al. (11). For case 2, the EEG and CSF analysis were normal, and she could not be diagnosed with probable anti-NMDAR encephalitis, as all three criteria should be fulfilled. Furthermore, the neurological autoantibody panel testing revealed a positive reaction to anti-NMDAR antibodies in CSF, which cause further diagnostic confusion. To our knowledge, this is the first study that shows the positive coexistence of anti-NMDAR antibodies and 14-3-3 protein in CSF of patients with anti-NMDAR encephalitis. The 14-3-3 test can be false-positive in several neurologic diseases characterized by acute and extensive neuronal damage, such as paraneoplastic neurologic disorders, autoimmune encephalitis associated with VGKC complex antibody (6, 7, 9, 12, 13). A previous study tested the CSF of 24 patients with anti-NMDAR encephalitis and found that none of them were positive for 14-3-3 protein (7). However, for CSF 14-3-3 protein detection in diagnosing sCJD, a systematic review reported an overall sensitivity of 92% and a specificity of 80% (12). A specificity of 80% in a disease with prevalence as low as that of sCJD means that most positive detection will represent false positives; therefore, the utility of the 14-3-3 test in the diagnosis of sCJD has its limitation, and it should be considered an adjunctive rather than diagnostic test for the diagnosis of prion diseases.

The current study had several limitations. First, the newly approved prion test, second-generation (RT-QuIC), was not performed due to the restriction on technique. RT-QuIC demonstrates a 92% sensitivity and 100% specificity for the diagnosis of sCJD (14). Second, we did not test the microtubule-associated protein tau due to the limitation of technique conditions. Tau protein is largely used as a surrogate CSF biomarker for sCJD, and it has shown comparable sensitivity and specificity to 14-3-3 (15). Third, due to the small number of cases, a prospective study is required to ascertain the frequency of 14-3-3 in autoimmune encephalitis.

## Conclusion

Anti-NMDAR encephalitis and sCJD are two different neurological disorders, and the clinical and investigative features should allow their differentiation in the majority of patients. These two cases illustrate the difficulty in determining the etiology of rapidly progressive dementia and that sometimes laboratory tests can be misleading. Patients suspected to have sCJD should be examined for the presence of NMDAR antibodies, even with the presence of 14-3-3 protein and MRI findings compatible with sCJD. Early consideration of immunomodulatory therapy is recommended, given that anti-NMDAR encephalitis is a treatable immune-mediated disorder, especially when titers of NMDAR antibodies are increased dramatically in patients with progressive encephalopathy.

## Data Availability Statement

The original contributions presented in the study are included in the article/supplementary materials, further inquiries can be directed to the corresponding author/s.

## Ethics Statement

The studies involving human participants were reviewed and approved by Clinical Trial and Biomedical Ethics Committee of West China Hospital of Sichuan University. The patients/participants provided their written informed consent to participate in this study. Written informed consent was obtained from the individual's legal guardian/next of kin for the publication of any potentially identifiable images or data included in this article.

## Author Contributions

XC, LW, JL, LC, and HS were involved in the conception, design, and data interpretation. LW, JL, LC, and JY were involved in the collection of data. XC and HS were involved in the drafting of the paper. All authors approved the final version.

## Conflict of Interest

The authors declare that the research was conducted in the absence of any commercial or financial relationships that could be construed as a potential conflict of interest.
